# Antibody Targeting of Cathepsin S Inhibits Angiogenesis and Synergistically Enhances Anti-VEGF

**DOI:** 10.1371/journal.pone.0012543

**Published:** 2010-09-02

**Authors:** Claire Ward, Diana Kuehn, Roberta E. Burden, Julie A. Gormley, Thomas J. Jaquin, Mihaela Gazdoiu, Donna Small, Roy Bicknell, James A. Johnston, Christopher J. Scott, Shane A. Olwill

**Affiliations:** 1 Drug Discovery, Fusion Antibodies Ltd, Belfast, United Kingdom; 2 School of Pharmacy, Queens University Belfast, Belfast, United Kingdom; 3 Schools of Immunity and Infection and Cancer Studies, College of Medicine and Dentistry, University of Birmingham, Birmingham, United Kingdom; 4 Centre for Infection and Immunity, Queens University Belfast, Belfast, United Kingdom; Bauer Research Foundation, United States of America

## Abstract

**Background:**

Angiogenesis is a key hallmark of tumourigenesis and its inhibition is a proven strategy for the development of novel anti-cancer therapeutics. An important aspect of early angiogenesis is the co-ordinated migration and invasion of endothelial cells through the hypoxic tumour tissue. Cathepsin S has been shown to play an important role in angiogenesis as has vascular endothelial growth factor (VEGF). We sought to assess the anti-angiogenic effect of Fsn0503, a novel cathepsin S inhibitory antibody, when combined with anti-VEGF on vascular development.

**Methodology/Principal Findings:**

Cathepsin S expression and secretion from endothelial cells was characterised using RT-PCR and western blotting. We further show that cathepsin S promotes pericellular hydrolysis of extracellular matrix components in the tumour microenvironment and facilitates endothelial invasion. The cathepsin S inhibitory antibody, Fsn0503, blocks extracellular proteolysis, inhibiting endothelial invasion and tube formation in cell-based assays. The anti-angiogenic effects of Fsn0503 were also shown *in vivo* where it significantly retarded the development of vasculature in human xenograft models. Furthermore, when Fsn0503 was combined with an anti-VEGF antibody, a synergistic inhibition of microvascular development was observed.

**Conclusions/Significance:**

Taken together, this data demonstrates that the antibody-mediated targeting of cathepsin S represents a novel method of inhibiting angiogenesis. Furthermore, when used in combination with anti-VEGF therapies, Fsn0503 has the potential to significantly enhance current treatments of tumour neovascularisation and may also be of use in the treatment of other conditions associated with inappropriate angiogenesis.

## Introduction

One of the hallmarks of tumour progression is the development of new blood vessels in order to supply the tumour with its metabolic requirements [1], [2]. Disruption of tumour angiogenesis has been extensively investigated to enable the development of novel anti-tumour strategies. For example, blocking tumour neovascularisation through abrogation of the vascular endothelial growth factor (VEGF) pathway with antibodies such as bevacizumab has proved therapeutically viable [3]–[5]. However, despite the clinical usefulness of these anti-VEGF strategies, a lack of efficacy, together with resistance and toxicity has been observed in some patients [6], [7]. Furthermore, anti-VEGF treatments have induced increased metastasis in animal models, highlighting the need for alternative anti-angiogenic strategies [8].

Recently the cysteine protease cathepsin S has been shown to play a key role in angiogenesis. Cathepsin S activity is normally restricted to the lysosomes of professional antigen presenting cells, mediating cleavage of the invariant chain from MHC class II complexes prior to antigen loading for presentation [9], [10]. However, in addition to adaptive immunity deficiencies, cathepsin S null mice also exhibit impaired endothelial microvessel development, suggesting a key role for this protease in angiogenesis [11]. Further studies have shown that cathepsin S is markedly up-regulated by endothelial cells during tumour angiogenesis [12], [13] and compellingly, in a murine pancreatic islet carcinoma model (RIP1-Tag2), cathepsin S knockout mice had a significant reduction in tumour-associated angiogenic switching and neovascularisation [14]. Taken together, these studies have highlighted the potential of targeting cathepsin S in the tumour microenvironment.

We have previously shown that the application of an inhibitory antibody to cathepsin S, Fsn0503, can block tumour development [15]. In this current study we demonstrate the mode of action of Fsn0503 towards endothelial cells and that it can be used in combination with an anti-VEGF antibody to synergistically block angiogenesis. This highlights the utility of targeting endothelial cell activation through more than one mechanism or pathway.

## Methods

### Cell culture

Human umbilical vein endothelial cells (HUVEC) (TCS Cellworks, Buckingham, UK) were grown in large vessel endothelial cell growth medium (TCS Cellworks) on 0.1% gelatin coated dishes up to passage 7. HMEC-1 cells [16] were maintained in MCDB-131 medium (Invitrogen, UK) supplemented with 10% fetal calf serum (FCS) (PAA Laboratories, Somerset, UK) epidermal growth factor (EGF, 10 ng/ml) (Roche, East Sussex, UK) and L-glutamine (10 mmol/L) (Invitrogen, UK). All cultures were maintained in a humidified environment at 37°C with 5% CO_2_.

### RT-PCR and Western blotting

HUVEC cells were stimulated with VEGF (Sigma, UK) (10 ng/ml) for 24 h or placed in a hypoxic chamber (0.1% oxygen) for 24 h and then used for RNA isolation or cell lysate preparation. Basal expression in HMEC-1 cells was also assessed. RNA was extracted using STAT 60 and cDNA was synthesised by using Im-Prom reverse transcription system (Promega) and used as the template for subsequent PCR. The following conditions were used: incubate at 95°C for 10 min and 40 cycles of 95°C for 30 secs, 55°C for 30secs and 72°C for 1 min followed by 70°C for 10min. PCR products were separated on a 1% agarose gel and photographed. Primers for the amplification of cathepsin S were forward ATGAAACGGCTGGTTTGTGTGC and reverse CTAGATTTCTGGGTAAGAGGG and for GAPDH forward ACCACAGTCCATGCCATCAC and reverse TCCACCACCCTGTTGCTGTA.

To prepare whole cell lysates, cell pellets were washed in PBS and lysed using standard RIPA buffer supplemented with a protease inhibitor cocktail (1∶50 dilution of lysis buffer). Samples were incubated for 20 minutes on ice prior to a 10 minute centrifugation at 13,000 rpm. Protein concentration was determined by BCA assay. Denatured samples were analysed by SDS-PAGE on 12% (w/v) polyacrylamide gels. Gels were transferred by semi-dry blotting onto nitrocellulose membrane, blocked with 3% dried milk before probing with 0.4 µg mouse anti-Cathepsin S antibody in 15mls PBS over night at 4°C. Following washes in PBS-T (PBS Tween), membranes were probed with goat anti-mouse-HRP (1∶3000) for 1 hr at room temperature. After a series of further washes with PBS-T, the membranes were developed using Chemiluminescent substrate (West Pico Chemiluminescent Substrate, Pierce) for 5 mins.

### Cathepsin S Activity assays

Cathepsin S activity was determined through the incubation of cell lysate and cell media samples in the presence of the fluorescently quenched tripeptidyl substrate Cbz-Val-Val-Arg-amino methyl coumarin (VVR-AMC, Merck Biosciences) as previously described [17]. Briefly, cell lysates were prepared using a 100 mmol/L sodium acetate lysis buffer containing 100mmol/L NaCl and 0.1% Triton X-100. A pre-incubation step was used to enhance substrate specificity. Before starting the assay, samples were incubated in 100mmol/L phosphate buffer (pH 7.5) for 1 hour at 37°C to inactivate non-cathepsin S activity, after which the pH was returned to pH 6.0 using 0.5mol/L of MES buffer. Assays were performed in triplicate in 96-well microtitre plates in the presence of 1 mmol/L dithiothreitol, 200 mmol/L ethylenediaminetetraacetate, and 100 µmol/L VVR-AMC in 200 mmol/L MES buffer (pH 6.0). The rate of substrate hydrolysis was monitored at 37°C every 60 seconds for 1 hour.

### Invasion assay

*In-vitro* invasion assays were performed as previously described [17]. Briefly, 8.0 µm polycarbonate membranes were coated with 1 mg/ml Matrigel placed in the wells of a 24 well plate (Cosatar). 2.5×10^5^ cells were seeded in the upper chamber of the invasion plate in serum free media in the presence of Fsn0503 or appropriate controls. The lower chamber was filled with media containing 10% FCS and an equimolar concentration of antibody or controls. Invasion plates were incubated at 37°C and 5% CO_2_ for 18 h. Non-invaded cells were removed and invaded cells were fixed in Carnoy's fixative for 15 min. After drying, the nuclei of invaded cells were stained with Hoechst 33258 (50 ng/mL) in PBS for 30 min, the membranes were washed, mounted and viewed with a Nikon eclipse TE300 fluorescent microscope. Each condition was performed in duplicate and 8 representative fields of view for each condition was imaged at magnification x20. Results were presented as mean number of cells invading per field of view. For the purpose of assessing inhibition by Fsn0503, the results were presented as a proportion of invasion relative to untreated control cells (100% cell invasion) as percentage ±SD cell invasion.

### Live cell proteolysis assay

Pre-cooled glass coverslips were coated with 25 µg/ml of quenched fluorescent substrate, DQ-Gelatin (Molecular Probes) in a 2% gelatin/2% sucrose solution in PBS and incubated on ice for 15 mins to solidify. 50,000 cells were seeded onto each coverslip and incubated at 37°C for 1 hr. The cells were then incubated in media with either Cathepsin S or isotype control antibodies (500 nmol/L) for 24 hrs. Fluorescent degradation products were observed using a Leica SP2 AOBS confocal microscope, with x40 oil immersion objective.

### CD34 staining

6–8 week old female BALB/c nude mice were implanted with 2×10^6^ HCT116 cells subcutaneously on either flank. On the day of implantation mice were randomly separated into treatment groups (n = 10). Mice were treated with 10 mg/kg isotype control or Fsn0503 cathepsin S antibody intraperitoneally 5 times per week for 4 weeks. Tumours were excised and formalin-fixed for histopathology. All animal experiments were carried out in accordance with the Animal (Scientific Procedures) Act 1986 and conformed to current UKCCCR guidelines under the project license PPL2560b issued by Dept of Health, Social Services and Public Safety, Castle Buildings, Belfast, Northern Ireland.

Tumour xenograft sections were stained for CD34 using the Vectastain ABC detection kit. Antigen retrieval was performed by microwaving in citrate buffer. Anti-mouse CD34 antibody (Abcam, MEC 14.7 clone, 2 ug/ml) and biotinylated anti-rat secondary antibody (vectorlabs) were used. Slides were counterstained with haematoxylin prior to mounting.

Sections from five mice were selected per treatment group, with 10 fields of view per section (0.29 mm^2^ each) randomly analyzed, excluding necrotic areas. The total number of CD34 positive cells, small vessels and large vessels were quantified using Nis Elements Br 2.30 software (Nikon, UK). For the purpose of vessel stratification, small vessels were classified as those below a 1500 µm^2^ cut-off while all others were classified as large vessels. The average vessel area was also assessed for each treatment group.

### Endothelial tube formation assay

12-well plates were coated with BD matrigel and incubated at 37°C for 1 h. 1.2×10^5^ HUVEC were plated per well in the presence of an isotype control or Fsn0503 cathepsin S antibody (400 nmol/L) in M199 media (Invitrogen) and incubated for 16 h. For combination with the anti-VEGF antibody, cells (1×10^5^) were plated in the presence of 10 ng/ml VEGF (Sigma, UK) and 250 ng/ml anti-VEGF antibody (Roy Bicknell) or 200 nM Fsn0503 or a combination of both. 4 fields of view were imaged (Nikon eclipse TS100 microscope) from duplicate wells and nodes with 1, 2, 3, 4 or more branches counted and the average calculated per field of view.

### MTT Assay

Cytotoxicity of Fsn0503 was determined using the MTT viability assay. Briefly, 5×10^3^ HUVECs were plated in a 96-well plate overnight. Fsn0503 or isotype control antibody (400 nM) was added to the cells and incubated at 37°C and 5% CO_2_ for 48 h. 20 µl of 5 mg/ml MTT (Thiazolyl Blue Tetrazolium Blue) was added in 200 µl of media for 4 h. The MTT reagent was removed and 200 µl of DMSO was added to dissolve the insoluble formazan crystals. Absorbance was read at 570 nm and results were expressed as the percentage cell viability relative to vehicle-only control. All tests were done in quintuplicate.

### Statistics

In vitro experiments were performed in triplicate, standard error of the mean and p values were calculated using students t-test and compared to the isotype control. Analysis of small versus large vessel number between treatment groups was measured using Pearson chi-squared test. A two tailed t-test was used to compare the mean vessel area between treatment groups (data was normalised prior to analysis) (*p<0.01; **p<0.001; ***p<0.0001).

## Results

### Pro-angiogenic stimuli promote the expression of cathepsin S in endothelial cells

**Figure 1:**
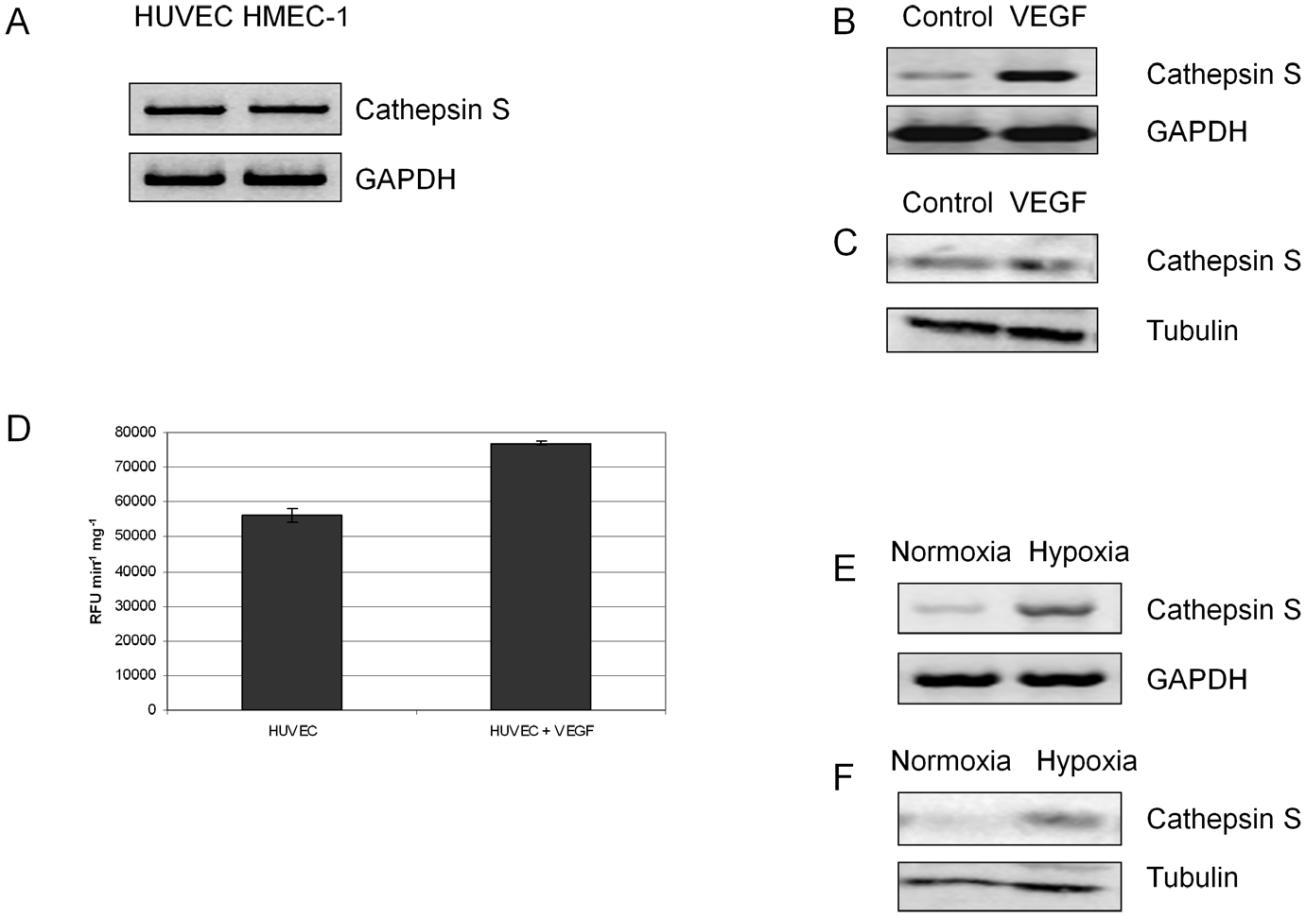
Cathepsin S expression and activity is up-regulated by pro-angiogenic stimuli. (**A**) Total RNA was isolated from endothelial cell lines (HUVEC and HMEC-1) and used to assess Cathepsin S mRNA by RT-PCR. GAPDH serves as a control. (**B,C**) Human recombinant VEGF (10 ng/ml) up-regulates cathepsin S mRNA and protein in HUVECs when assessed by RT-PCR and western blot using GAPDH and tubulin as respective controls (**D**) Human recombinant VEGF (10 ng/ml) stimulates Cathepsin S-like activity, as assessed by the cleavage of fluorigenic substrate, Cbz-Val-Val-Arg-AMC, by 37% in HUVEC cell lysates. (**E,F**) To demonstrate the effect of hypoxia on Cathepsin S up-regulation total RNA and protein was isolated from HUVEC endothelial cells (grown in normal or hypoxic conditions) and examined by RT-PCR and western blot. GAPDH and tubulin serves as a control.

Endothelial cathepsin S has been shown to play an important role in the facilitation of tumour angiogenesis [11], [12]. In the present study we have analysed the expression of cathepsin S from endothelial cell lines (HUVEC and HMEC-1) at the mRNA level (Fig. 1A). The results confirmed expression of the protease in these cells and we further demonstrated its secretion into the growth media (data not shown). As VEGF is a major pro-angiogenic growth factor and up-regulated in many tumours; we stimulated growth factor starved HUVECs with VEGF and observed an up-regulation of the cathepsin S at the mRNA and protein levels (Fig. 1B, C). Moreover, VEGF stimulation of the HUVECs increased the secreted levels of cathepsin S-like activity by 37% (Fig. 1D). The production of VEGF is normally as a result of hypoxia in rapidly developing tumours and its presence predisposes to a more malignant phenotype [18]. Therefore we subsequently examined the alteration in cathepsin S expression in hypoxic HUVECs and observed increased expression (Fig. 1E, F). This is in agreement with gene expression analysis showing that endothelium under ‘tumour’ conditions (hypoxia, low pH, low glucose) expressed a high level of cathepsin S transcript while it was absent in the same cells under normal conditions (normoxia 4% oxygen, normal pH, normal glucose) (Herbert JMJ and Bicknell R, unpublished data). Collectively, these results confirm the over-expression of endothelial cathepsin S in pro-angiogenic environments.

### Fsn0503 blocks endothelial cell invasion and tube formation through inhibition of ECM degradation

**Figure 2:**
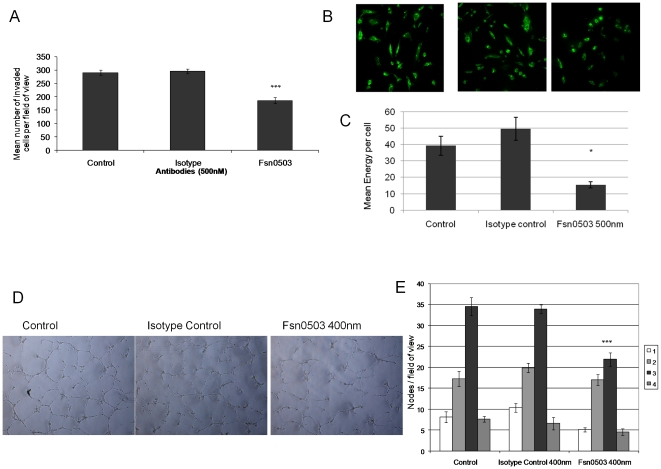
Fsn0503 demonstrates anti-angiogenic effects *in vitro*. (**A**) Fsn0503 attenuates HUVEC invasion using a modified Boyden chamber assay at 500 nM. (**B**) Fsn0503 (500 nM) inhibits HUVEC degradation of quenched fluorescent substrate DQ gelatin. Cells treated with Fsn0503 or controls were analysed for the presence of fluorescent degradation products by confocal microscopy (representative images shown). (**C**) A reduction of 70% of DQ gelatin degradation was observed when quantified by mean energy per cell (p = 0.03) (5 fields per assay). (**D**) Fsn0503 (400 nM) inhibits HUVEC tube formation compared to isotype control (representative images shown). (**E**) Tube formation was assessed by counting nodes with 1, 2, 3, or more branches and the average calculated per field of view. The number of nodes with 3 or more branches, which are indicative of normal tube formation, were significantly reduced in Fsn0503 treated samples (p<0.01).

Given that we have previously observed that secreted cathepsin S enhances the invasion of tumour cells through the extracellular matrix, inducing a pericellular zone of proteolysis around the cells [15], we wished to ascertain whether a similar effect was seen in endothelial cells. Using the cathepsin S inhibitory antibody, Fsn0503 [15], we evaluated its application in attenuating HUVEC invasion through the ECM *in vitro*. In these studies we observed a significant block in cell invasion in the presence of Fsn0503, of up to 37% at 500 nmol/L (Fig. 2A).

To confirm that the block in invasion was a result of inhibition of ECM degradation we examined the ability of the endothelial cells to degrade fluorescently labeled ECM. In the absence of treatment or in presence of an isotype antibody, the generation of fluorescence was clearly evident around the periphery of the endothelial cells (Fig. 2B). These effects are in agreement with the secretion of the protease and its pericellular proteolytic activity. However, this fluorescence was significantly reduced (70%, p = 0.03) (Fig. 2B,C) in the presence of the Fsn0503 antibody (500 nmol/L), indicating the blocking of ECM hydrolysis through the inhibition of the extracellular cathepsin S.

The functional anti-angiogenic significance of these findings was revealed using endothelial tube formation assays. In early angiogenesis, after proliferation and migration into the interstitial stroma, endothelial cells connect with each other forming tube-like structures, a process that can be replicated *in vitro*. In agreement with our earlier findings, Fsn0503 inhibited HUVEC tube branching and formation, significantly reducing the number of nodes containing 3 or more branches by over 30% at 400 nmol/L (p<0.01) (Fig. 2D,E). These findings are consistent with the attenuation of endothelial cell localised invasion and movement. To confirm that the effects of the antibody were anti-invasive and not as a result of cellular toxicity, we examined the viability of HUVECs incubated with Fsn0503 using MTT assays and found no effect on cell viability at 400 nmol/L over a period of 48 h (data not shown).

### Fsn0503 blocks tumour blood vessel development *in vivo*

**Figure 3:**
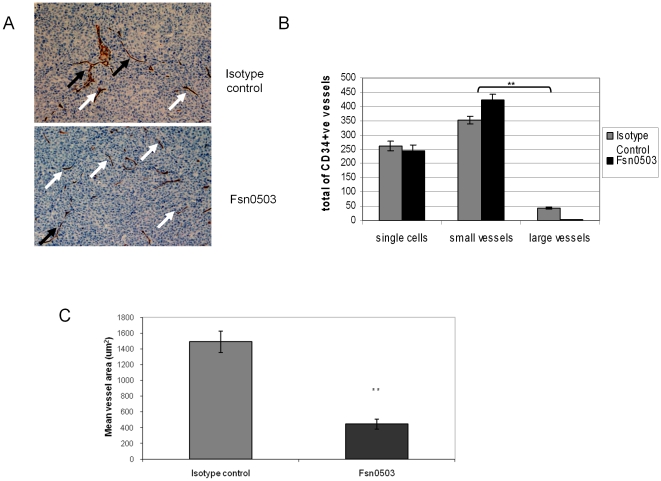
Fsn0503 inhibits angiogenesis *in vivo*; CD34 Immunohistochemical analysis of Fsn0503 treated tumors. (**A**) Pattern of small vessel (white arrows) and large vessel (black arrows) distribution in isotype control and Fsn0503 treated tumors (10 mg/kg 5 times a week for 4 weeks) (20×). (**B**) Analysis of total vessel number as characterized by CD34 staining shows that Fsn0503 caused an increase in small vessel number and a significant decrease in the number of large vessels (p<0.001). (**C**) A significant reduction is observed in the mean vessel area of tumours treated with Fsn0503 (p<0.001).

Previously we have observed impaired infiltration of tumour-associated cells into HCT116 colorectal carcinoma xenografts on treatment with Fsn0503 [15]. In the present study, we established HCT116 xenografts, and then treated with either isotype control or the Fsn0503 cathepsin S antibody before subsequently quantifying developing neovasculature using the endothelial marker CD34. Analysis of total CD34 positive cells revealed an increase in the number of small vessels and a significant decrease in the number of large vessels (p<0.001) in tumours treated with Fsn0503 (Fig. 3A,B). A significant reduction in the mean vessel area in tumours treated with Fsn0503 (p<0.001) was also observed (Fig. 3C). Collectively, these findings support the role of cathepsin S in promotion of neovascularisation, and highlight it as a target for the development of novel anti-angiogenic drugs such as the inhibitory antibody Fsn0503.

### Inhibition of cathepsin S and VEGF synergistically blocks new vessel development

**Figure 4:**
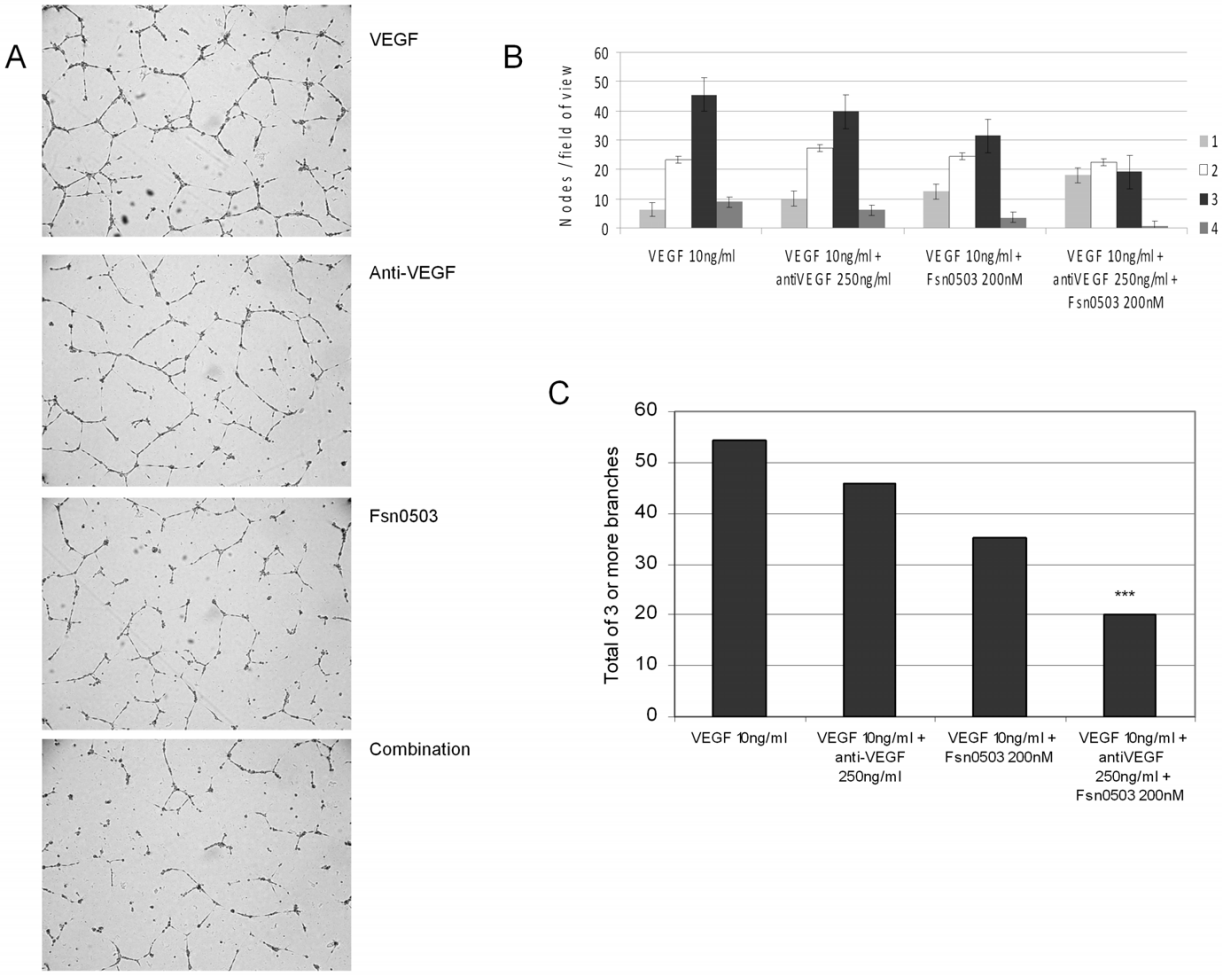
Fsn0503 in combination with an anti-VEGF antibody synergistically inhibit angiogenesis *in vitro*. (**A**) Representatives tube assay images of VEGF control, anti-VEGF antibody treated, Fsn0503 treated and the combination of anti-VEGF antibody and Fsn0503 which shows the greatest disruption of tube formation. (**B, C**) Tube formation was assessed by counting nodes with 1, 2, 3, or more branches and the average calculated per field of view. The number of nodes with 3 or more branches, which are indicative of normal tube formation were assessed to demonstrate efficacy. Quantification of tube formation shows the single agents reducing the number of nodes with 3 or more branches and the combination synergistically reducing the number of nodes with 3 or more branches and increasing the number of single branches.

On the basis of the potent *in vitro* and *in vivo* anti-angiogenic properties elicited by Fsn0503 when used as a monotherapy, we next examined if Fsn0503 could enhance the efficacy of an anti-VEGF antibody. HUVEC tube formation was measured in the presence of both an anti-VEGF antibody and the Fsn0503 cathepsin S antibody alone or in combination. Fsn0503 caused a reduction in the number of nodes containing 3 or more branch points, characteristic of a disrupted network. As anticipated, a similar pattern was observed with the anti-VEGF antibody (Fig. 4A). However the combination, of Fsn0503 and anti-VEGF induced further disruption of the tube network with a synergistic reduction of nodes containing 3 or more branch points and an increase in the number of single branch points (Fig. 4B,C).

## Discussion

Cathepsin S is a cysteine protease that has been shown to play a key role in the development of tumour neovasculature [11]–[13]. Normally, Cathepsin S has limited tissue distribution, found primarily in lysosomes of professional antigen presenting cells. However, it is up-regulated and secreted into the tumour microenvironment by a number of tumour-associated cell types, including endothelial cells. Previously, we have described the development of an inhibitory antibody, Fsn0503 [15], and now demonstrate its mode of action through inhibition of matrix degradation and invasion of endothelial cells resulting in the inhibition of tube formation *in vitro*. In agreement with these findings, a significant reduction in the mean vessel area and the number of large vessels was observed in Fsn0503 treated xenografts. Furthermore, the application of Fsn0503 in combination with a VEGF antibody produced a synergistic anti-angiogenic effect in the HUVEC tube formation assay.

Currently, targeting the VEGF pathway is one of the main focuses of anti-angiogenic treatment. VEGF is a prominent pro-angiogenic factor promoting endothelial cell proliferation and survival and is up-regulated by hypoxia. It is expressed in approximately 30–60% of most solid tumours and up to 100% of renal cell carcinoma [19]. Increased expression is associated with a poor prognosis in a range of human tumours including colorectal cancer [20]. Successful anti-VEGF therapies include bevacizumab, which has been approved for the treatment of colorectal cancer in combination with chemotherapy [21], and sorafenib, a VEGF receptor tyrosine kinase inhibitor approved for metastatic renal cell carcinoma [2]. Unfortunately, as many patients treated with anti-angiogenic agents/VEGF inhibitors eventually relapse, the benefit from VEGF targeted therapy can be relatively short. Moreover, studies have also shown when anti-VEGF therapies are withdrawn, the anti-tumor effect is rapidly lost due to the un-affected tumor extracellular matrix [22]. Furthermore, indications that VEGF inhibitors attenuate primary tumor growth but yet promote invasiveness and metastasis has raised concerns [23]. These potential limitations to current therapies support a two-pronged approach to therapy which comprises an anti-VEGF agent with an anti-invasive compound such as Fsn0503. Indeed, we have shown here that combination of an anti-VEGF antibody and a cathepsin S antibody can generate synergistic effects, probably as a result of inhibiting both pro-angiogenic signaling and cell motility.

Cathepsin S is one of a family of 11 cysteine proteases, many of which have been shown to be up-regulated in tumours [24]. Furthermore, distinct causative roles in tumorigenesis for three of these proteases, namely cathepins B, L and S have been revealed in a murine model of pancreatic islet carcinomas [14]. In this model, cathepsins B and S were both found to similarly promote tumour angiogenesis. However, of these two enzymes, cathepsin S may represent a more appropriate anti-angiogenic therapeutic target as it has a more restricted tissue expression pattern than cathepsin B. Cathepsin S also distinguishes itself from other cathepsins by exhibiting catalytic activity over a broad range of pH (4.5–8.0) and has increased stability over other cathepsins in the extracellular environment [24], [25]. Indeed, these findings are consistent with other findings in cathepsin S null mice which demonstrate impaired microvessel development [11] and in tumour models, impaired tumour angiogenesis [12]. Interestingly, this later study also showed that extracellular cathepsin S may mediate its pro-angiogenic function not only through the remodeling of ECM, but in the release of pro-angiogenic factors and degradation of anti-angiogenic factors derived from ECM. We here show that endothelial cells, under tumour-like conditions can increase their expression of cathepsin S. These findings are in agreement with microarray analysis of endothelial cDNA from murine hepatocarcinomas which showed that cathepsin S was the more significantly up-regulated protease during tumour angiogenesis [13]. Taken collectively with our own findings, it suggests that the specific targeting of extracellular cathepsin S will have clinical utility for blocking tumour angiogenesis. As the pathological cathepsin S is in the extracellular environment, this makes it particularly amendable to antibody-based inhibitors such as Fsn0503.

Further potential combination treatment strategies using Fsn0503 may be considered in the light of these results. Previously, the application of a broad spectrum experimental cathepsin probe, JPM-OEt, has been used with chemotherapy to further enhance efficacy of treatment [26]. Therefore the combination of a clinically applicable inhibitor such as Fsn0503 and chemotherapies may produce similar synergistic effects to the drug combinations that have been used here, particularly in inhibiting the spread of invasive and malignant tumours.

In conclusion, we have demonstrated that Fsn0503 has clear anti-angiogenic properties that modulate endothelial cells in a manner distinct from anti-VEGF treatments. We believe that the combination of anti-angiogenics with different modes of action can benefit the treatment of tumour neovascularisation and may also be of use in the treatment of other conditions where inappropriate angiogenesis is occurring.
